# Socioeconomic Disparities and Influenza Hospitalizations, Tennessee, USA

**DOI:** 10.3201/eid2109.141861

**Published:** 2015-09

**Authors:** Chantel Sloan, Rameela Chandrasekhar, Edward Mitchel, William Schaffner, Mary Lou Lindegren

**Affiliations:** Brigham Young University, Provo, Utah, USA (C. Sloan);; Vanderbilt University School of Medicine, Nashville, Tennessee, USA (C. Sloan, R. Chandrasekhar, E. Mitchel, W. Schaffner, M.L. Lindegren)

**Keywords:** health care disparities, influenza, minority health, spatial analysis, Census Bureau, Tennessee, United States

## Abstract

We examined population-based surveillance data from the Tennessee Emerging Infections Program to determine whether neighborhood socioeconomic status was associated with influenza hospitalization rates. Hospitalization data collected during October 2007–April 2014 were geocoded (N = 1,743) and linked to neighborhood socioeconomic data. We calculated age-standardized annual incidence rates, relative index of inequality, and concentration curves for socioeconomic variables. Influenza hospitalizations increased with increased percentages of persons who lived in poverty, had female-headed households, lived in crowded households, and lived in population-dense areas. Influenza hospitalizations decreased with increased percentages of persons who were college educated, were employed, and had health insurance. Higher incidence of influenza hospitalization was also associated with lower neighborhood socioeconomic status when data were stratified by race.

## Abstract 2

High rates of poverty, low education, and female single-parent households are associated with these hospitalizations.

Influenza causes annual outbreaks that result in >200,000 hospitalizations and 3,300–49,000 deaths annually in the United States (*1*). Children <2 years of age, persons >65 years of age, pregnant women, and those with underlying health conditions are at greater risk for developing serious complications (e.g., pneumonia) from influenza and are at greater risk for hospitalization and death. Despite continuing vaccine and treatment interventions, the public health effects of annual influenza epidemics remain substantial.

Although patient-level risk factors for severity of influenza have long been identified, attention is being directed towards reporting neighborhoods and contextual and environmental characteristics that increase risk for adverse health outcomes and that are independent of patient-level attributes (*2*). Geographic-based measures include physical, social, and economic characteristics of neighborhoods, such as poverty level, education, residential segregation, psychosocial stress, unemployment, inadequate transportation, social networks, distance to medical facilities, access to prevention and treatment services, insurance status, environmental exposures, and housing and density characteristics. Disparities in health outcomes likely result from a combination of factors that influence an individual’s exposures, risk behaviors, susceptibility, treatment options, and social contextual factors (*3**–**5*). However, rarely are these measures collected through population-based surveillance systems. Previous work investigating influenza disparities showed a strong positive correlation between influenza hospitalization rates and geographic areas of high poverty and household crowding (*6**,**7*).

We analyzed population-based influenza hospitalization surveillance data from the Tennessee Emerging Infections Program (EIP) (*8**,**9*) to identify potential disparities in influenza hospitalization rates in Middle Tennessee according to neighborhood-level measures of socioeconomic status (SES). Understanding disparities in influenza hospitalization rates is a priority for the EIP as necessary to reduce illness and death from annual influenza epidemics.

## Methods

### The Study Setting and Population

**Figure 1:**
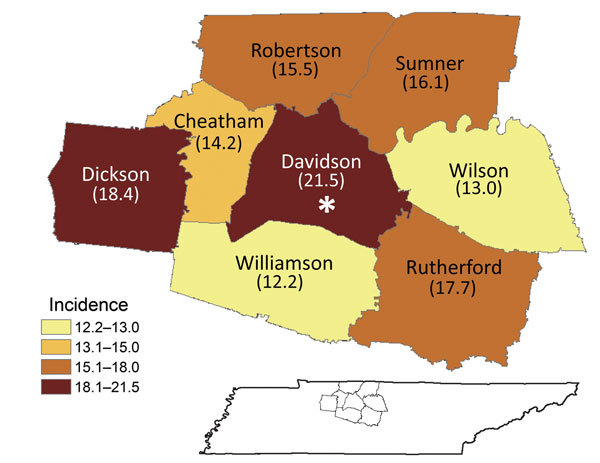
Average annual incidence of influenza hospitalizations, by county, Middle Tennessee, USA, October 2007–April 2014. Asterisk indicates location of the city of Nashville.

Using the Tennessee EIP Influenza Hospitalization Surveillance Network, we analyzed data collected during the 2007–08 through 2013–14 influenza seasons. As part of the Influenza Hospitalization Surveillance Network, the Tennessee EIP conducts population-based surveillance for laboratory-confirmed influenza hospitalizations in 8 counties located in Middle Tennessee, which includes the city of Nashville, located in Davidson County, and its bordering suburban and rural counties: Wilson, Rutherford, Williamson, Dickson, Cheatham, Robertson, and Sumner (Figure 1). The population size of the catchment area is ≈1,557,000 persons. 

Laboratory confirmation for influenza virus infection was determined by reverse transcription PCR, viral culture, direct or indirect fluorescent antibody staining, or rapid antigen testing. Influenza testing was ordered at the discretion of the treating clinicians. Those hospitals without onsite PCR capacity were encouraged to send specimens to the Tennessee Department of Health Laboratory Services for reverse transcription PCR confirmation. Surveillance for laboratory-confirmed influenza hospitalization was reviewed and determined to be exempt by the Human Subjects Review Board at the Centers for Disease Control and Prevention and by the Human Research Protection Program at the Vanderbilt University School of Medicine. 

Information about demographic characteristics, underlying conditions, clinical outcomes, and antiviral treatment was collected from medical record review by trained reviewers who used a standard questionnaire. Surveillance was conducted annually during the influenza season (October–April). During the influenza A(H1N1) pandemic of 2009–10, surveillance continued throughout the summer. We included race in the analysis but did not stratify by ethnicity because of low numbers identified as Hispanic ethnicity. 

Each participant’s home address was geocoded to a latitude and longitude point by using ArcMap version 10.0 (*10*). Most (94%) addresses were geocoded successfully; those that could not be geocoded to rooftop accuracy were excluded. Each home address was assigned to a Tennessee census tract on the basis of location.

### Census Data

We used the assigned census tracts to extract data from the 2010 US Census and from the 2007–2011 American Community Survey. For each tract, census data included tract population, percent below poverty, health insurance status, education, employment, and percentages of female head of household and household crowding. We also calculated population density per square mile by using census population totals and areas calculated within ArcMap. When possible, we categorized sociodemographic variables according to previously published standards by the Harvard Geocoding Project (*11*). Table 1 shows the categorization of the major sociodemographic factors from the American Community Survey.

**Table 1:** Average annual crude and age-standardized incidence rates and relative rates of influenza hospitalization by demographic and neighborhood measures, Middle Tennessee, USA, October 2007–April 2014*

Characteristic	Hospitalizations, no. (%), N = 1,743	Crude incidence (95% CI)	Age-standardized incidence (95% CI)	Rate ratio (95% CI)	Rate difference (95% CI)	RII†
Individual-level data†						
Sex						NA
M	775 (44.5)	15.1 (14.0–16.2)	16 (14.9–17.2)	NA	NA	
F	968 (55.5)	18.0 (16.8–19.1)	17.8 (16.7–19.0)	1.1 (1.0–1.2)	1.8 (0.2–3.4)	
Race§						NA
White	1,242 (73.4)	15.3 (14.5–16.2)	15.2 (14.4–16.1)	NA	NA	
African American	418 (24.7)	24.7 (22.4–27.1)	27.4 (24.8 30.3)	1.8 (1.6–2.0)	12.2 (9.4–15.0)	
Other	31 (1.8)	4.4 (2.8–5.9)	4.0 (2.5–6.5)	0.3 (0.2–0.4)	−11.2 (−13.1 to –9.3)	
Age, y						NA
<5	207 (11.9)	28.3 (24.4–32.2)	NA	NA	NA	
5–17	98 (5.6)	5.3 (4.3–6.4)	NA	NA	NA	
18–49	470 (27.0)	9.6 (8.7–10.4)	NA	NA	NA	
50–64	398 (22.8)	20.7 (18.7–22.8)	NA	NA	NA	
≥65	570 (32.7)	51.7 (47.4–55.9)	NA	NA	NA	
Neighborhood-level data‡						
% Below poverty						
<5.0	266 (15.3)	11.4 (10.0–12.8)	11.5 (10.1–13.0)	NA	NA	2.9 (2.5–3.5)
5.0–9.9	374 (21.5)	14.2 (12.8–15.6)	13.9 (12.5–15.4)	1.2 (1.1–1.4)	2.4 (0.5–4.4)	
10.0–19.9	475 (27.3)	17.3 (15.7–18.8)	16.8 (15.3–18.4)	1.5 (1.3–1.7)	5.3 (3.3–7.4)	
≥20.0	628 (36)	24.9 (22.9–26.8)	25.7 (23.7–27.8)	2.2 (2.0–2.5)	14.2 (11.8–16.7)	
% College education						
15.0–24.9	16 (0.9)	38.8 (19.8–57.7)	47.3 (23.9–92.1)	NA	NA	0.5 (0.4–0.7)
25.0–39.9	326 (18.7)	21.5 (19.2–23.9)	21.4 (19.1–23.9)	0.5 (0.1–1.7)	−25.9 (−53.7 to 1.8)	
≥40.0	1,401 (80.4)	16.1 (15.3–17)	16.1 (15.2–16.9)	0.3 (0.1–1.9)	−31.3 (−58.9 to −3.6)	
% Employed						
<50.0	1,122 (64.4)	19.3 (18.2–20.4)	18.9 (17.8–20.1)	NA	NA	0.6 (0.5–0.7)
50.0–65.9	605 (34.7)	14.1 (12.9–15.2)	14.4 (13.3–15.6)	0.8 (0.7–0.9)	−4.5 (−6.1 to −2.9)	
≥66.0–74.9	16 (0.9)	12.6 (6.4–18.8)	15.8 (8.4–27.7)	0.8 (0.5–1.4)	−3.2 (−11.9–5.5)	
% Female HH						
<20.0	637 (36.5)	12.7 (11.8–13.7)	12.7 (11.7–13.7)	NA	NA	3.2 (2.7–3.8)
20.0–39.9	531 (30.5)	17.2 (15.7–18.6)	17.2 (15.7–18.7)	1.4 (1.2–1.5)	4.5 (2.7–6.3)	
40.0–59.9	340 (19.5)	23.0 (20.6–25.4)	22.7 (20.3–25.3)	1.8 (1.6–2.0)	10.0 (7.4–12.6)	
≥60.0	235 (13.5)	34.9 (30.5–39.4)	36.0 (31.5–41.0)	2.8 (2.5–3.2)	23.3 (18.6–28.1)	
Household crowding, persons/room)						
<5.0	1,514 (86.9)	16.5 (15.7–17.3)	16.4 (15.5–17.2)	NA	NA	1.9 (1.5–2.5)
5.0–9.9	176 (10.1)	20.0 (17.0–23.0)	21.6 (18.4–25.1)	1.3 (1.1–1.5)	5.2 (1.9–8.6)	
≥10.0	53 (3.0)	27.5 (20.1–34.9)	26.9 (20.0–35.6)	1.6 (1.2–2.2)	10.5 (3.1–17.9)	
Population density, persons/mi^2^						
0–<200	259 (14.9)	14.8 (13.0–16.6)	14.0 (12.3–15.8)	NA	NA	1.8 (1.5–2.2)
200–700	273 (15.7)	13.8 (12.2–15.5)	13.7 (12.1–15.5)	1.0 (0.8– 1.2)	−0.3 (−2.6– 2.1)	
≥700	1,211 (69.5)	18.6 (17.5– 19.6)	18.7 (17.7– 19.8)	1.3 (1.2–1.5)	4.7 (2.7–6.8)	
% Medical insurance						
50–74.9	200 (11.5)	22.5 (19.4–25.6)	24.1 (20.8–27.8)			0.5 (0.3–0.6)
>75.0	1,543 (88.5)	16.5 (15.7–17.3)	16.4 (15.6–17.2)	0.7 (0.5–0.8)	−7.8 (−11.3 to −4.3)	

*HH, head of household; RII, relative indexes of inequality; NA, not applicable. †RII is calculated as the exponent of the slope of a Poisson regression model by using incidence rate as the outcome variable and the proportion of the population in that socioeconomic group as the predictor variable. The RII can be interpreted similarly to an incidence rate ratio that compares those in the quantitatively highest category with those in the lowest categorization. For example, an RII of 2.5 would indicate a 150% increase in risk when those in the quantitatively highest category are compared with those in the lowest (such as the <49.9% category being compared with the **≥**66.0–74.9 category for patients employed). A low RII (with CIs) <1 would indicate decreased risk. An RII was not calculated for variables marked NA because they do not have a readily available ordinal variable by which to compare lowest and highest socioeconomic status.  ‡Sex, race, and age characteristics use individual-level data from surveillance; neighborhood-level characteristics use data from the American Community Survey. §The number of patients with available race data was 1,691.

Overall population density was calculated by dividing the total number of persons by the number of square miles in each census tract (*12*). We further categorized population densities into 3 categories: <200 persons/square mile, 201–700 persons/square mile, and ≥700 persons/square mile. These categories were selected because they differentiated geographic areas that were predominantly rural, suburban, or urban in our population (Technical Appendix Figure 1) 

### Statistical Analysis

The data were analyzed by using R version 3.0.1 (http://www.r-project.org/) and SAS version 9.3 (SAS Institute, Inc., Cary, NC, USA). We calculated the Spearman’s rank correlation coefficient (r_s_) between each variable to determine which ones were likely to provide redundant results. The most highly correlated variables were single-parent household and female head of household (r_s_ = 0.96), percentage below poverty and single-parent household (r_s_ = 0.76), and overall population density and population density of children <5 years of age (r_s_ = 0.96; Technical Appendix Figure 2). Percentage of white residents and population density were negatively correlated (r_s_ = −0.66), as were percentage below poverty and median income (r_s_ = −0.89). Percentage of single-parent households, median income, and population density of children are not presented in the results because of the high correlation among these variables. Percentage below poverty was selected instead of median income because Krieger et al., in a comparison of different SES measures, found percentage below poverty to be the most robust indicator of neighborhood poverty (*11*). 

We calculated the average annual incidence of influenza hospitalizations per 100,000 person-years during the 7-year period as the proportion of persons hospitalized in the catchment area per 100,000 persons per year. We also calculated the age-standardized rate ratio (RR), rate difference (RD), relative index of inequality (RII), concentration curve (CC), and its associated concentration index (CIndex) for each census variable. The RII is used as a measure of the strength of the influence of SES on health inequality. RII is calculated as the exponent of the slope of a Poisson regression model by using incidence rate as the outcome variable and the proportion of the population in a socioeconomic group as the predictor variable. The RII can be interpreted similarly to an incidence RR by comparing those in the quantitatively highest category with those in the lowest category. For example, an RII of 2.9 would indicate a 190% increase in risk if those in the highest categorization are compared with those in the lowest. CCs were used to discern whether results were biased because of cutoffs used for variable categorization. The CC is a graph of the cumulative percentage of cases versus the cumulative percentage of the population distribution of the census tract variable. If no health disparities are present, the curve will fall on the diagonal. A curve above the diagonal indicates that patients are concentrated in the highest risk category. What is shown qualitatively by the CC can be summarized quantitatively by the CIndex. It is computed as twice the area between the curve and the diagonal line. A negative CIndex shows a disparity in influenza hospitalizations regarding levels of the census variable that indicate low SES (*13*). If no census variable-related inequality is present, the CIndex is 0.

## Results

During the influenza seasons from 2007–08 through 2013–14, a total of 1,743 persons were hospitalized with confirmed influenza in the Middle Tennessee catchment area. The number of persons hospitalized ranged from 61 during the 2011–12 season to 590 during the 2013–14 season. The observed frequency of influenza hospitalizations was in accordance with those reported by other surveillance sites. Low rates were observed nationally during the 2011–12 season (*14*).

Women had a higher age-standardized incidence rate of hospitalizations (17.8/100,000 population; 95% CI 16.7–19.0) compared with that for men (16.0/100,000 population; 95% CI 14.9–17.2; Table 1). This finding was consistent over the study period. The highest incidence by age group was for those >65 years of age (51.7/100,000 population; 95% CI 47.4–55.9), compared with an incidence rate of 5.3/100,000 population (95% CI 4.3–6.4) for those 5–17 years of age, the group with the lowest rate (Table 1). Children <5 years of age had incidence rates of 28.3/100,000 population (95% CI 24.4–32.2). African Americans had an age-standardized incidence rate of 27.4/100,000 population (95% CI 24.8–30.3), compared with a rate for whites of 15.2/100,000 population (95% CI 14.4 –16.1). African Americans had higher rates than whites for all 7 seasons investigated (Technical Appendix Figure 3). 

**Figure 2:**
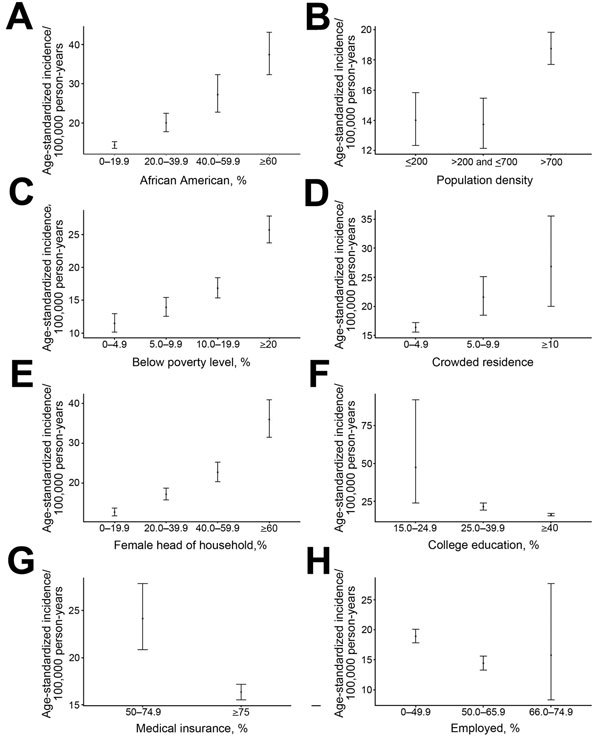
Age-standardized incidence of influenza hospitalizations by census tract socioeconomic variables, Middle Tennessee, USA, October 2007–April 2014. Variables were linked to the American Community Survey. A) Incidence by percentage of African Americans. B) Incidence by population density (<200 persons/mi^2^ [rural]; >200–<700 persons/mi^2^ [suburban]; >700 persons/mi^2^ [urban]). C) Incidence by percentage living below poverty level. D) Incidence by level of crowded housing (persons per room). E) Incidence by percentage with female head of household. F) Incidence by percentage with college education. G) Incidence by percentage with medical insurance. H) Incidence by percentage employed. Error bars indicate 95% CIs.

Crude and adjusted rates of influenza hospitalization for each variable studied are shown in Table 1. For census tracts with increasing percentages of the population employed, insured, and college educated, rates of influenza hospitalizations decreased (RII 0.5, 95% CI 0.4–0.7; RII 0.6, 95% CI 0.5–0.7; and RII 0.5, 95% CI 0.3–0.6, respectively) (Table 1). Figure 2 shows age-standardized rates for variables by levels of SES. For census tracts having the lowest percentage of persons below the poverty level (i.e., <5% of the population), the age-standardized incidence rate of influenza hospitalization was 11.5/100,000 population (95% CI 10.1–13.0; Figure 2). For those tracts with the highest percentage below poverty (>20% of the population), the incidence rate was 25.7/100,000 population (95% CI 23.7–27.8; Figure 2). RRs increased with increasing percentage of the population living below poverty. Compared with the <5% below poverty tracts, tracts with 5%–9.9%, 10%–19.9% and >20% of persons living below poverty had RRs of 1.2, 1.5, and 2.2, respectively (RII 2.9, 95% CI 2.5–3.5). The RD also increased according to percentage of the population living below poverty (2.4, 5.3, and 14.2, respectively, for tracts with 5%–9.9%, 10%–19.9% and >20% living below poverty). 

In addition, rates increased from 12.7/100,000 population (95% CI 11.7–13.7) for tracts with <20% female heads of household to 36.0/100,000 population (95% CI 31.5–41.0) for tracts with >60% female heads of household (RII 3.2, 95% CI 2.7–3.8). The RD increased from 4.5 for tracts with 20%–39.9% female heads of households to 10.0 for tracts with 40%–59.9% female heads of households to 23.3 for >60% female heads of households. Household crowding was also associated with increased risk for influenza hospitalization (RII 1.9, 95% CI 1.5–2.5). 

Urban census tracts (i.e., those with population densities >700/square mile) had consistently higher influenza hospitalization rates (18.7/100,000 person-years) than did tracts with lower population densities (13.7 and 14.0/100,000 person-years in suburban [201–700 persons per square mile] and rural [<200 persons/square mile] areas; RII 1.8, CI 1.5–2.2) (Table 1). This trend was consistent across influenza seasons.

**Figure 3:**
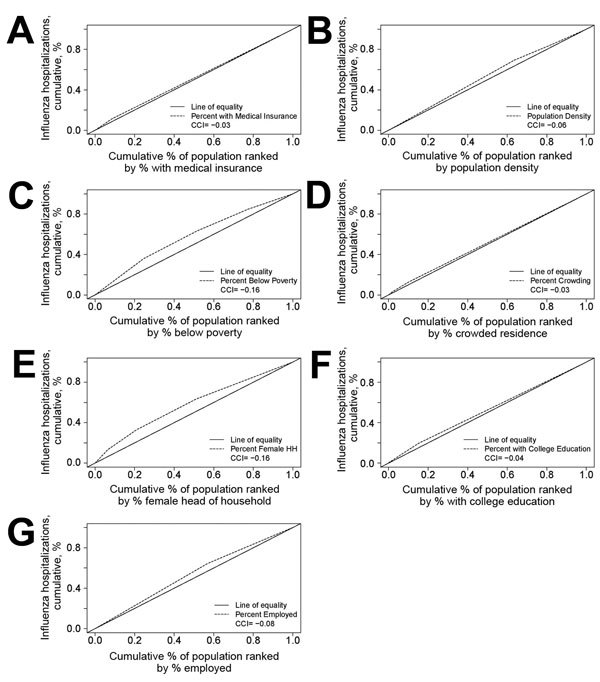
Concentration curves of neighborhood-level disparities in influenza hospitalizations, Middle Tennessee, USA, October 2007–April 2014. Figures show the divergence of cumulative incidence of hospitalizations for factors from the American Community Survey from the line of equality. In the absence of disparities, the dotted and dashed lines would entirely overlap. Cumulative percentage of the population hospitalized for influenza is shown for A) percentage of the population with medical insurance; B) population density; C) percentage of the population below poverty; D) percentage of the population with different levels of residential crowding; E) percentage of the population with female-headed households; F) percentage of the population with a college education; and G) percentage of the population employed. CCI, concentration curve index.

Although every variable showed some deviation from the line of equality (Figure 3), percent below poverty and percent female head of household each had a CIndex of −0.16 (Figure 3), indicating strong disparities. The percent employed variable also showed a disparity in hospitalizations with a CIndex of −0.08.

We calculated age-standardized incidence by race for selected characteristics (Table 2). A comparison of white patients residing in neighborhoods with >20% of persons living below poverty with those living in areas with <5% below poverty resulted in an RII of 2.5 (95% CI 2.0–3.1); the RII for the same comparison for African Americans was 3.3 (95% CI 2.2–4.8). Approximately two thirds of African Americans hospitalized with influenza during the study period resided in census tracts with the highest percentage of persons living below poverty (i.e., >20%). We also calculated incidence for age and race for household crowding and female head of household. The RII for African Americans by percentage of female heads of household was 3.6 (95% CI 2.5–5.1), compared with 2.4 for whites (95% CI 2.0–3.0). Overall age-standardized rates for household crowding were similar for each race group (Table 2).

**Table 2:** Average annual age-standardized and race-stratified incidence of influenza hospitalizations, by neighborhood percentage of households below poverty, household crowding, and percentage of households with female head of household, Middle Tennessee, USA, October 2007–April 2014*

Characteristic	Hospitalizations, no. (%)	Age-standardized annual incidence (95% CI)	Rate ratio	Rate difference	RII†
White, n = 1,242					
% Below poverty					
<5.0	233 (18.8)	11.0 (9.6–12.5)			2.5 (2.0–3.1)
5.0–9.9	320 (25.8)	13.6 (12.2–15.2)	1.2 (1.1–1.4)	2.7 (0.6–4.7)	
10.0–19.9	374 (30.1)	16.3 (14.7–18.1)	1.5 (1.3–1.7)	5.3 (3.1–7.5)	
≥20.0	315 (25.4)	23.0 (20.5–25.7)	2.1 (1.8–2.4)	12.0 (9.1–14.9)	
Household crowding‡					
<5.0	1,113 (89.6)	14.8 (13.9–15.7)			1.9 (1.3–2.8)
5.0–9.9	99 (8.0)	19.1 (15.5–23.2)	1.3 (1.1–1.6)	4.3 (0.4–8.1)	
10.0+	30 (2.4)	26.7 (17.9–38.9)	1.8 (1.3–2.6)	11.9 (2.2–21.6)	
% Female head of household					
<20.0	556 (44.8)	12.4 (11.4–13.5)			2.4 (2.0–3.0)
20.0–39.9	423 (34.1)	16.9 (15.3–18.6)	1.4 (1.2–1.5)	4.5 (2.6–6.4)	
40.0–59.9	190 (15.3)	20.7 (17.8–24.0)	1.7 (1.4–1.9)	8.2 (5.1–11.4)	
60.0+	73 (5.9)	32.3 (25.1–41.3)	2.6 (2.1–3.3)	19.9 (12.2–27.5)	
African American, n = 418					
% Below poverty					
<5.0	20 (4.8)	17.8 (10.7–28.4)			3.3 (2.2–4.8)
5.0–9.9	40 (9.6)	15.9 (11.2–22.6)	0.9 (0.5–1.6)	−1.8 (−11.4 to 7.7)	
10.0–19.9	79 (18.7)	21.7 (17.1–27.3)	1.2 (0.8–1.9)	4.0 (−5.4 to 13.3)	
≥20.0	279 (66.7)	34.6 (30.6–39.0)	1.9 (1.5–2.5)	16.8 (7.8–25.8)	
Household crowding†					
<5.0	339 (81.1)	26.3 (23.5–29.3)			1.8 (1.1–2.8)
5.0–9.9	64 (14.6)	33.6 (25.2–44.4)	1.3 (1.0–1.7)	7.3 (−2.1 to 16.8)	
10.0+	15 (3.6)	41.3 (22.7–71.1)	1.6 (0.9–2.7)	15.0 (−6.6 to 36.7)	
% Female head of household					
<20.0	49 (11.7)	14.8 (10.9–19.9)			3.6 (2.5–5.1)
20.0–39.9	77 (18.4)	20.8 (16.1–26.8)	1.4 (1.0–1.9)	6.0 (−0.6 to 12.6)	
40.0–59.9	139 (33.3)	31.6 (26.5–37.4)	2.1 (1.7–2.6)	16.7 (10.0–23.5)	
60.0+	153 (36.6)	40.0 (33.8–46.9)	2.7 (2.2–3.3)	25.1 (17.5–32.8)	

*Rates within ethnic subpopulations (i.e., Hispanic) were not calculated because of low numbers for these groups. RII, relative index of inequality. †RII is calculated as the exponent of the slope of a Poisson regression model by using incidence rate as the outcome variable and the proportion of the population in that socioeconomic group as the predictor variable. The RII can be interpreted similarly to an incidence rate ratio that compares those in the quantitatively highest category with those in the lowest categorization. For example, an RII of 2.5 would indicate a 150% increase in risk when those in the quantitatively highest category are compared with those in the lowest (such as the <49.9% category being compared with the **≥**66.0–74.9 category for percentage of patients employed). A low RII with CIs <1 would indicate decreased risk.  ‡Household was evaluated for number of persons per room.

## Discussion

Area-based measures of disparities in SES were strongly associated with incidence of influenza hospitalization in Middle Tennessee. Increasing incidence of influenza hospitalization was associated with increasing proportion of the population living below poverty or having female-headed households and with increasing population density and household crowding. Decreasing incidence of influenza hospitalizations was associated with increasing percentages of the population having medical insurance, employment, and college education. RDs also consistently increased with increased percentages of persons living below poverty, of female-headed households, and of household crowding. These associations were consistent throughout each of the 7 influenza seasons studied. Increasing incidence with decreasing SES was also found within each racial group. Among individual-level characteristics, older age, African American race, and female sex were associated with increased incidence of influenza hospitalization. The choice of concentration curves as the main measurement of disparities indicated that neighborhood socioeconomic indicators were robust in their influence on disparities in influenza hospitalization.

Our findings that neighborhood SES disparities influence influenza hospitalizations rates extends conclusions found in other studies (*3**,**6**,**7**,**15**,**16*). Population-based influenza hospitalization surveillance data from Connecticut showed that increasing hospitalization rates for both adults and children were associated with decreasing SES measures and increasing household crowding (*6**,**7*). The similar findings in these 2 population-based surveillance systems in different US geographic locations, a highly populated state in the Northeast and a more rural state in the Southeast, support the robustness of these associations. Other studies have also identified neighborhood social and physical characteristics, including housing conditions and environmental exposures, as risk factors for asthma and influenza hospitalization (*6**,**17**–**20*). Charland et al. reported that communities with increasing prevalence of obesity, less physically active populations, and lower fruit and vegetable consumption had higher rates of influenza-related hospitalizations (*21*).

We incorporated 4 distinctive measures of socioeconomic disparities (RR, RD, RII, and CIndex) into the statistical analysis that builds on the work of Krieger (*22*) in measuring the effects of health disparities on influenza hospitalization in Tennessee health outcomes. We also constructed CCs, graphic representations of disparities. Although the RR and RD are traditionally reported in such analyses and are easy to interpret, they are sensitive to the values used in categorization of the socioeconomic variable. In contrast, the RII and CIndex are measures that reflect the experiences of the entire population and are sensitive to the distribution of the population across socioeconomic groups. Any CIndex with a value <0 indicates disparity (*13*).

Surveillance systems have usually not collected individual-level SES data but often use surrogate measures (e.g., race) to monitor health disparities. These surrogate measures have been inadequate to quantify SES inequalities in health. Area-based measures are the only currently available means to understand health inequities in population-based surveillance systems and may be uniquely relevant for monitoring the role of neighborhood in SES health inequities. Furthermore, the geospatial distribution of infectious diseases and area-based risk factors might be used to design, target, monitor, and assess public health programs, including prevention interventions for influenza. Age and underlying conditions of persons are currently used as the basis for targeted vaccination strategies. However, because area-based measures are strong risk factors for severe influenza, neighborhoods may become major targets for future preventive interventions.

This study has several limitations. First, data from population-based influenza hospitalization represent those who sought care and were tested for influenza by their clinician, and testing practices likely varied across hospitals in the catchment area. However, these data are consistently used each year by the Centers for Disease Control and Prevention to evaluate the severity of influenza and to determine persons at risk in real time during the influenza season. Second, we did not assess differences in influenza vaccination status among patients because data on vaccination coverage by census tract were not available, and the number of reported vaccinations on EIP case report forms was very low. Finally, neighborhood SES may not apply to specific individual-level SES characteristics and may not be the same for different persons. That is, neighborhood characteristics evaluated in this study may not well characterize individual persons living in those neighborhoods. However, these variables offer insight into the role of neighborhood in determining influenza health outcomes. We have defined neighborhoods as census tracts, although nearby neighborhoods may also influence health outcomes and disparities. 

In summary, increasing rates of hospitalizations in Middle Tennessee were associated with increasing percentages of the population living below poverty, having female heads of households, living in densely populated areas, and living in crowded household conditions. Decreasing hospitalization rates were seen in areas with increasing percentages of the population with health insurance, college education, and employment. The well-tested procedures for incorporating neighborhood-level data into health studies described by the Harvard Geocoding Project (*11*), along with the application of infrequently used. CIndexes and CCs implemented in this study have shown the importance of measuring neighborhood-level SES disparities in determining health outcomes, such as incidence of influenza hospitalization. These population-based data from Tennessee reinforce the association of area-based measures of SES with incidence of influenza hospitalization and emphasize the important role that neighborhood socioeconomics play in explaining rates described here. The study also suggests that, because neighborhood characteristics are strongly associated with hospitalization rates, they should be considered when designing targeted prevention strategies such as vaccination programs.

**Technical Appendix.** Population density of Middle Tennessee, USA, based on 2010 US Census, and correlations between neighborhood-level variables and age-standardized incidence of influenza hospitalization by season for October 2007–April 2014.
