# Supplementary material for: Socioeconomic Disparities and Influenza Hospitalizations, Tennessee, USA
Source: Emerg Infect Dis. 2015 Sep;21(9):1602–10. doi: 10.3201/eid2109.141861 (PMC4550146; doi:10.3201/eid2109.141861)
Supplement: Supplementary file 1 — Technical Appendix. Population density of Middle Tennessee, USA, based on 2010 US Census, and correlations between neighborhood-level variables and age-standardized incidence of influenza hospitalization by season for October 2007–April 2014. [file 14-1861-Techapp-s1.pdf]

Article DOI: http://dx.doi.org/10.3201/eid2109.141861

# Socioeconomic Disparities and Influenza Hospitalizations in Tennessee, USA

## Technical Appendix

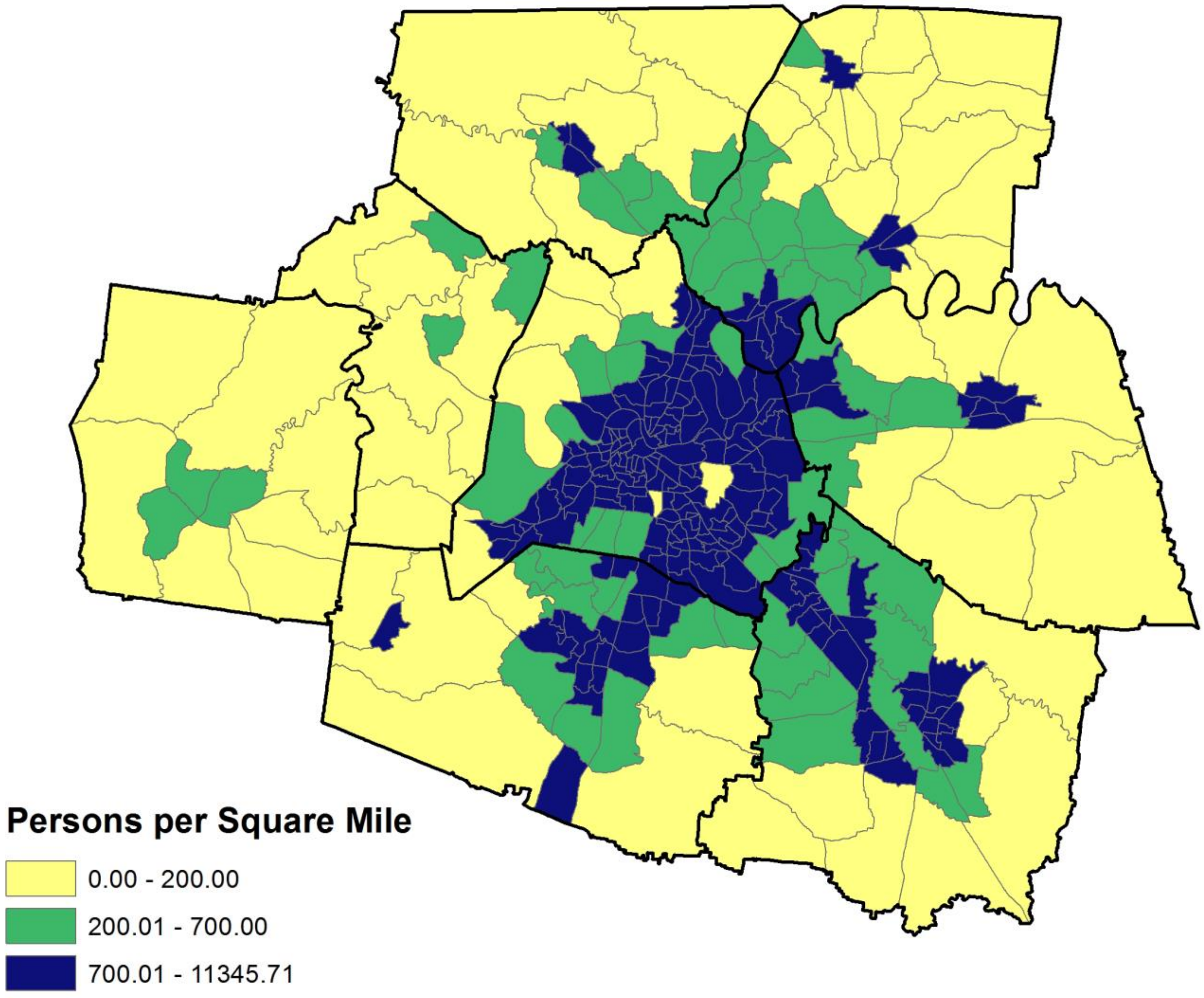


**Technical Appendix Figure 1.** Population density of Middle Tennessee, USA: persons per square mile. Density was calculated by dividing US Census totals per tract (2010) by the area calculated using ArcMAP version 10.0 (*1*).

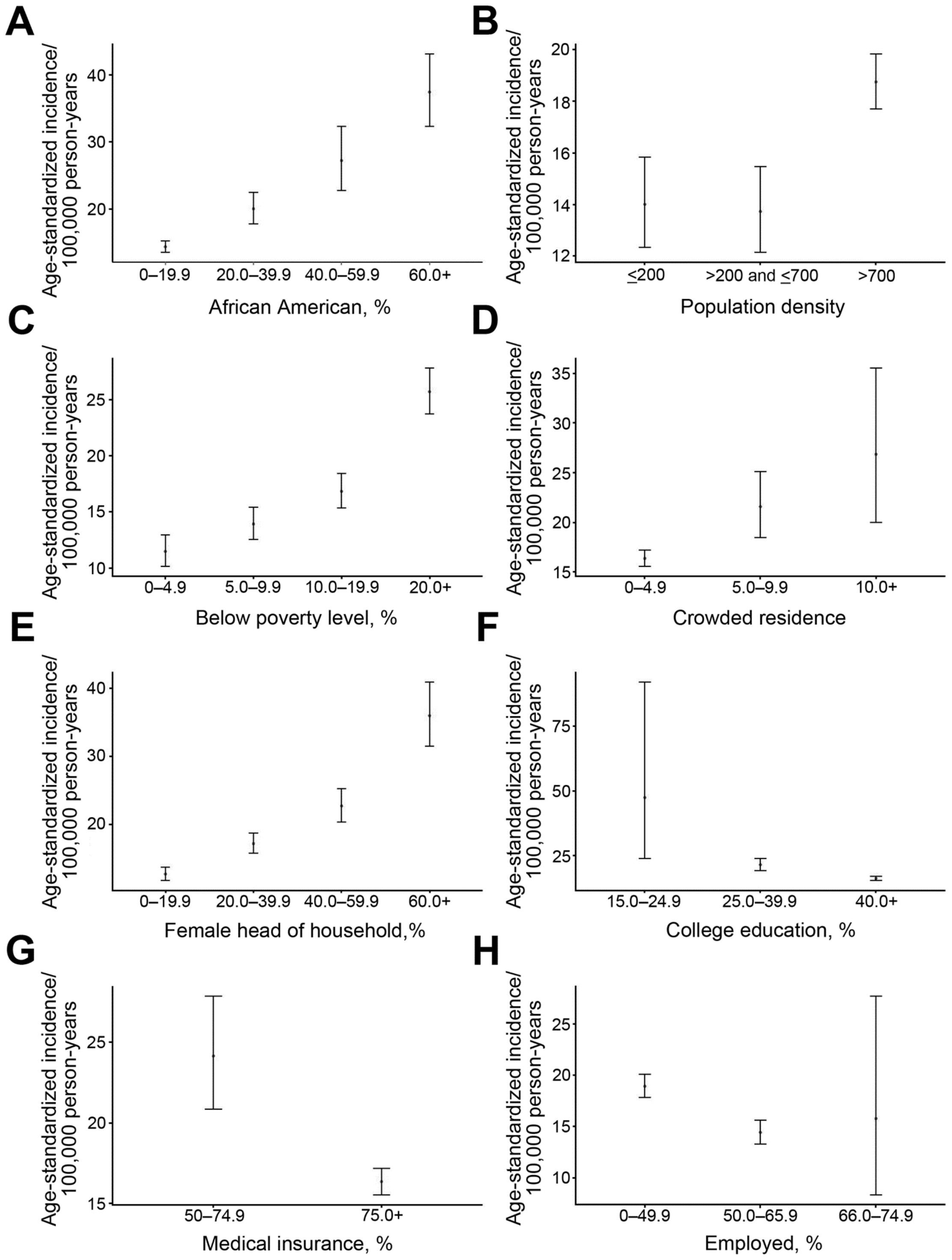


**Technical Appendix Figure 2.** Correlations between neighborhood-level variables, Tennessee, USA. Plot shows Spearman's pair-wise correlations between each neighborhood variable used in the analysis. Shown are smoothed trends in the bottom left, distributions of the individual variables down the diagonal line, and strength of the correlations to the right of the diagonal by number and font size.

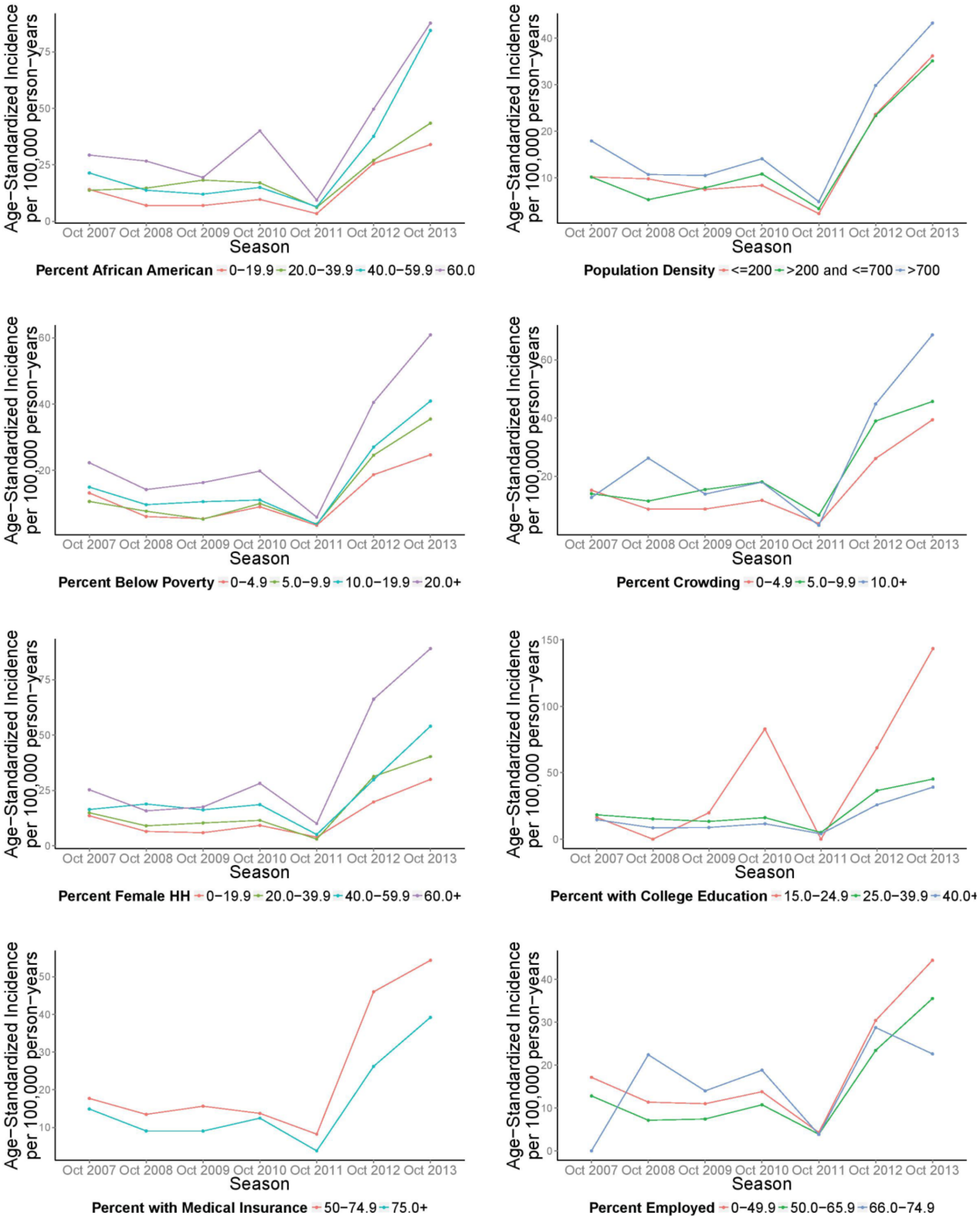


**Technical Appendix Figure 3.** Age-standardized incidence of influenza hospitalization by season for 8 neighborhood-level variables, Middle Tennessee, USA, October 2007–April 2014.

**Reference**

1. Krieger N, Chen JT, Waterman PD, Rehkopf DH, Subramanian S. Painting a truer picture of US socioeconomic and racial/ethnic health inequalities: the Public Health Disparities Geocoding Project. Am J Public Health. 2005;95:312–23. PubMed http://dx.doi.org/10.2105/AJPH.2003.032482
